# Supplementary material for: ARHGAP45 controls naïve T‐ and B‐cell entry into lymph nodes and T‐cell progenitor thymus seeding
Source: EMBO Rep. 2021 Mar 15;22(4):e52196. doi: 10.15252/embr.202052196 (PMC8024898; doi:10.15252/embr.202052196)
Supplement: Supplementary file 1 — Expanded View Figures PDF [file EMBR-22-e52196-s003.pdf]

Expanded View Figures

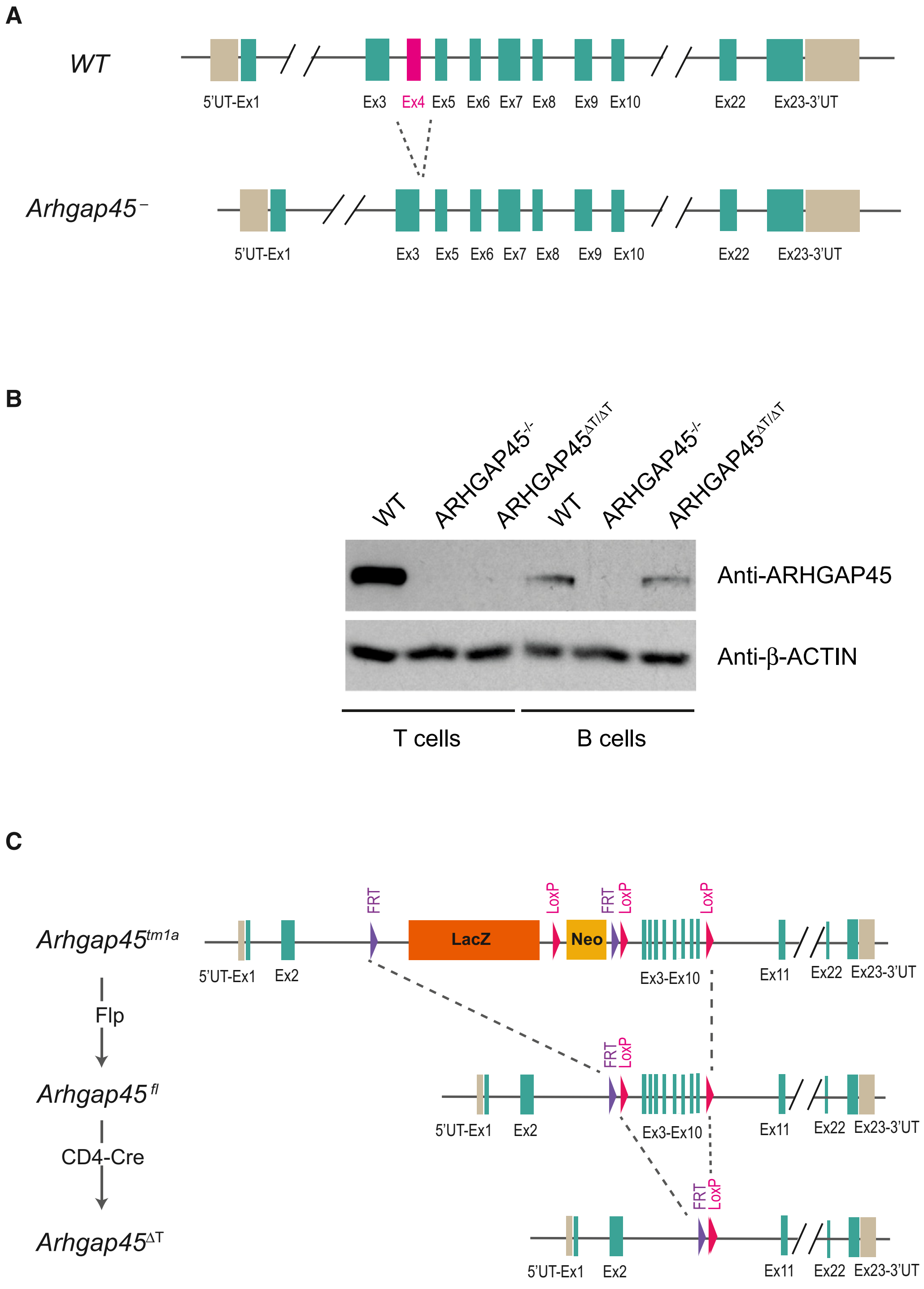

Figure EV1.

© 2021 The Authors

Figure EV1. Generation of mutant mouse lacking ARHGAP45, or expressing a *lox*P-flanked *Arhgap*45 allele.

A Schematic representation of the WT *Arghap45* and *Arghap45*⁻ alleles. Exons 1-23 are shown and numbered and the 5' and 3'UTR shown as gray box. The deletion engineered in the *Arghap45*⁻ allele encompasses exon 4 (http://www.ensembl.org/Mus_musculus/Transcript/Summary?db=core;g=ENSMUSG00000035697;r=10:80016653-80031472;t=ENSMUST00000099501).

B Immunoblot analysis of equal amounts of total lysates of thymocytes (left panel) and of B and T cells (right panel) purified from WT, *Arhgap45*$^{-/-}$, and *Arhgap45*$^{\Delta T/\Delta T}$ mice probed with anti-ARHGAP45 and anti-β-Actin (loading control). Molecular weights are shown on the left. Results are representative of two experiments.

C Schematic representation of the *Arhgap45*^{tm1a}, *Arhgap45*^{fl}, and *Arhgap45*$^{\Delta T}$ alleles. See "Generation of mice with a *lox*P-flanked *Arhgap*45 allele and of mice conditionally deprived of ARHGAP45 in T cells" in Materials and Methods. Exons 1–23 are shown and numbered and the 5' and 3'UTR shown as gray boxes.

Source data are available online for this figure.

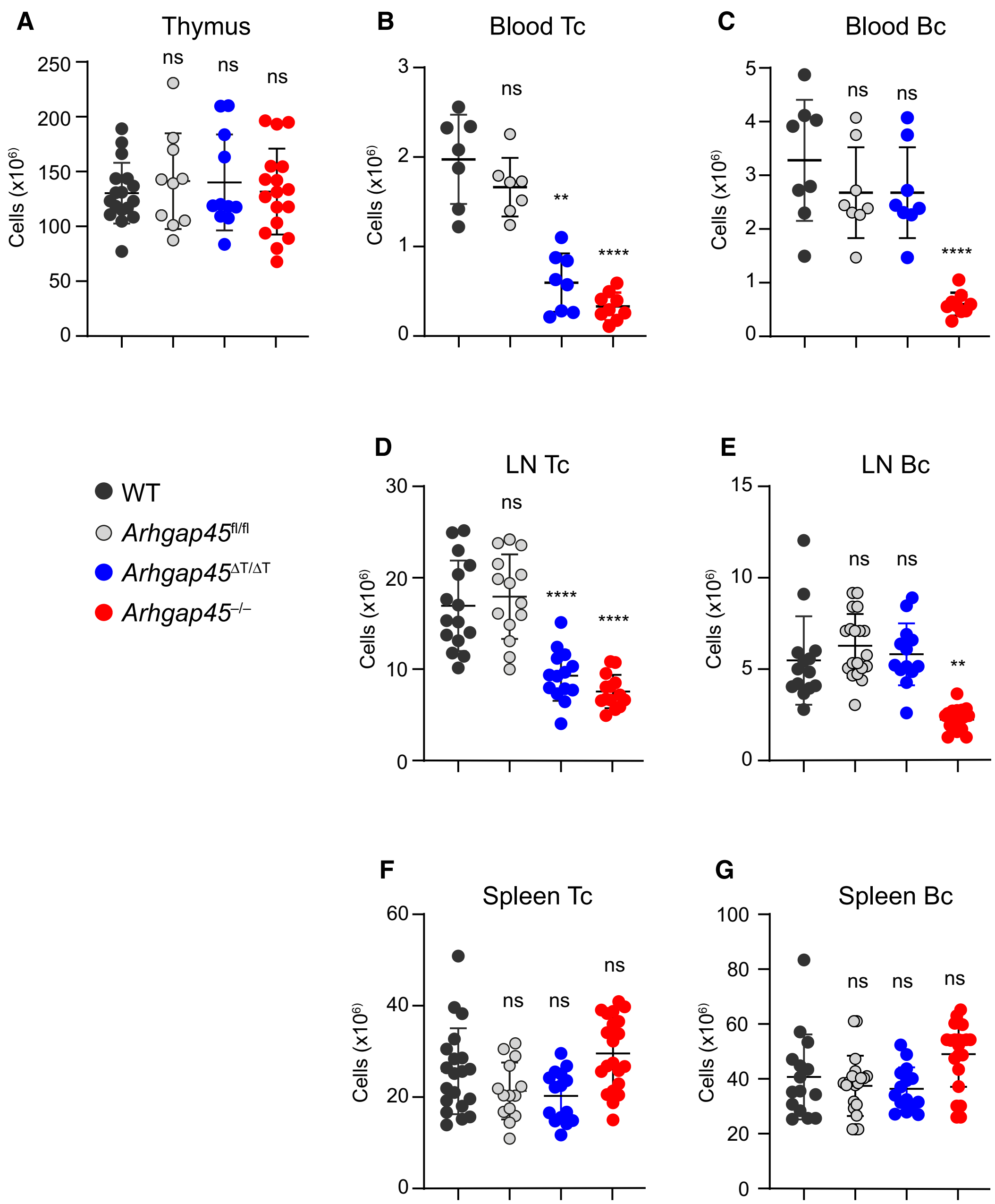

Figure EV2.

◀

Figure EV2. Numbers of B and T cells in the specified organs of WT mice and of mice deficient in ARHGAP45 (*Arhgap45*$^{-/-}$), or expressing a *lox*P-flanked *Arhgap*45 allele prior to (*Arhgap45*$^{fl/fl}$) or after (*Arhgap*45$^{\Delta T/\Delta T}$) crossing with CD4-*Cre* transgenic mice.

A Cellularity of thymus from the specified mice.
B Numbers of T cells found in the blood of the specified mice.
C Numbers of B cells found in the blood of the specified mice.
D Numbers of T cells found in the LNs of the specified mice.
E Numbers of B cells found in the LNs of the specified mice.
F Numbers of T cells found in the spleen of the specified mice.
G Numbers of B cells found in the spleen of the specified mice.

Data information: Each dot corresponds to a mouse and the mean and SD are indicated. Data are representative of three independent experiments involving each a total of 8–16 mice. A one-way Anova test was used to compare each mouse model against WT mouse controls. The resulting probability is indicated above each model. ns, non-significant, **$P \leq 0.002$, ****$P \leq 0.0001$.

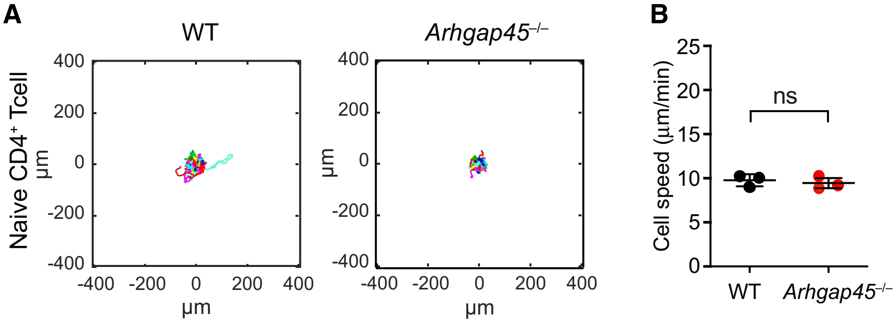

Figure EV3. Naïve T cells do not polarize and migrate on ICAM-1 coated surface in absence of added chemokine.

A Analysis of migration patterns of WT and *Arghap45*$^{-/-}$ naive T cells on 2D surface coated with ICAM-1. Each track represents the migratory path of individual WT and *Arghap45*$^{-/-}$ naive T cells recorded over 466 s in a single field of view. Trajectories were plotted to a common starting point, and > 200 T cells were recorded per plot using time-lapse microscopy at 10× magnification.
B Average cell speed of WT cell (black) and *Arghap45*$^{-/-}$ naive T cells (red). Three independent experiments were performed involving more than 200 cells and each dot corresponds to the mean of the speed in one given experiment. The speed of 10 µm/min correspond to Brownian motion, which means that cells are not motile (two-tailed Student's *t*-test).

 © 2021 The Authors

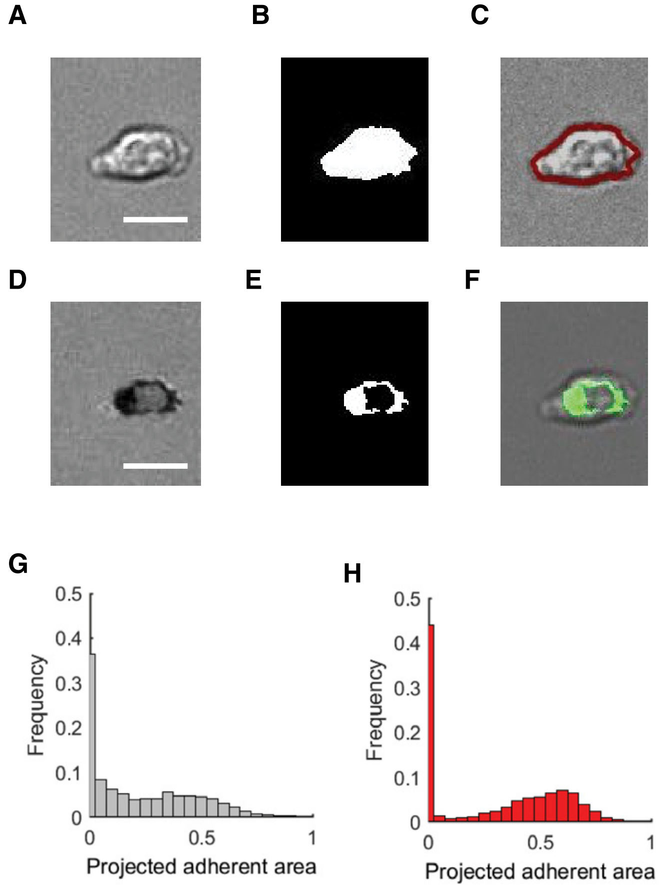

Figure EV4. Analysis of the projected adhesion area of WT and *Arghap45*$^{-/-}$ naive T cells on 2D surface coated with ICAM-1 CCL21.

A–F Processing of images from RICM microscopy to infer projected adhesion area. The projected area of cells is extracted from bright field images (A), which are binarized (B) to extract the contour in red (C). The area of adhesion fingerprint is assessed from RICM images (D) that are inverted and binarized (E) to extract the area of the contact zone in green (F). To illustrate image processing, the final image of the migration sequence shown in Fig 6C has been used. Scale bar: 10 μm.

G, H Histograms of instant projected adherent area for WT (G) and ARHGAP45$^{-/-}$ (H) naive T cells on 2D surface coated with ICAM-1 and CCL21.

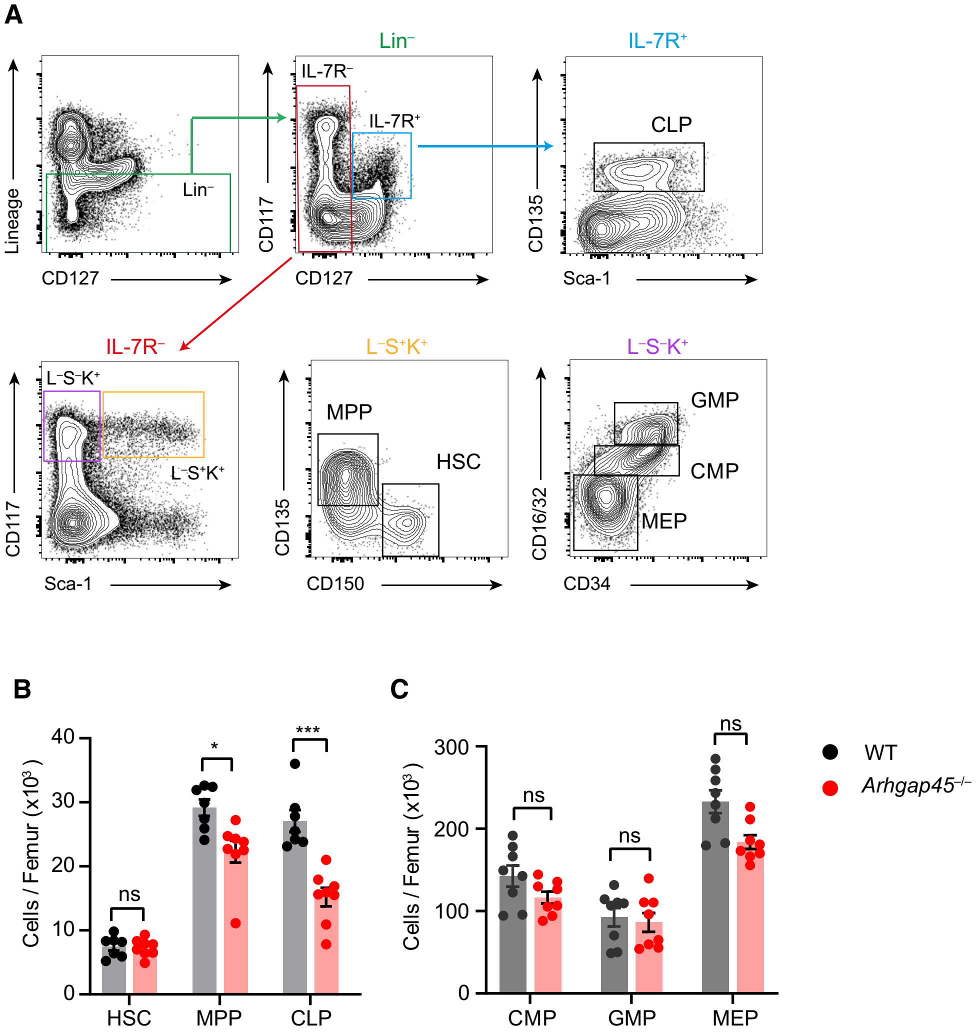

Figure EV5. Analysis of hematopoietic progenitors in the BM of WT and *Arhgap45*$^{-/-}$ mice.

A Gating strategy for the specified BM hematopoietic cell progenitors as described in Cordeiro Gomes *et al* (2016). HSC: hematopoietic stem cells, MPP: multipotent progenitors, CLP: common lymphoid progenitors, CMP: common myeloid progenitors, GMP: granulocyte and monocyte progenitors, and MEP: megakaryocyte and erythroid progenitors.

B Numbers of HSC, MPP and CLP per femur of WT and *Arhgap45*$^{-/-}$ mice.

C Numbers of CMP, GMP and MEP per femur of WT and *Arhgap45*$^{-/-}$ mice.

Data information: Each dot corresponds to a mouse and the mean and SD are indicated. Data are representative of three independent experiments. *$P \leq 0.01$, ***$P \leq 0.001$; unpaired Student's *t*-test.

 © 2021 The Authors
