# Supplementary material for: ARHGAP45 controls naïve T‐ and B‐cell entry into lymph nodes and T‐cell progenitor thymus seeding
Source: EMBO Rep. 2021 Mar 15;22(4):e52196. doi: 10.15252/embr.202052196 (PMC8024898; doi:10.15252/embr.202052196)
Supplement: Supplementary file 5 — Movie EV2 [file EMBR-22-e52196-s010.zip › MovieEV2/MovieEV2_legend.docx]

**Movie EV2** depicts *Arhgap45*^–/–^ activated T cells crawling on glass substrates coated with ICAM-1. The movie corresponds to the superposition of microscopy images taken in bright field mode. An image was recorded every 10 s at a 20 x magnification and the movie consists of 63 time frames.
