# Supplementary material for: ARHGAP45 controls naïve T‐ and B‐cell entry into lymph nodes and T‐cell progenitor thymus seeding
Source: EMBO Rep. 2021 Mar 15;22(4):e52196. doi: 10.15252/embr.202052196 (PMC8024898; doi:10.15252/embr.202052196)
Supplement: Supplementary file 7 — Movie EV4 [file EMBR-22-e52196-s005.zip › MovieEV4/MovieEV4_Legend.docx]

**Movie EV4** depicts *Arhgap45*^–/–^ naïve T cells crawling on glass substrates coated with ICAM-1 and CCL21. The movie corresponds to the superposition of microscopy images taken in bright field mode (grey) and in reflection interference contrast microscopy (RICM) mode (green). RICM images were binarized and intensity inverted to display the adhesion footprint in bright green. An image was recorded every 2 s at a 63 x magnification, and the movie consists of 400 time frames.
