# Supplementary figures and images for: ARHGAP45 controls naïve T‐ and B‐cell entry into lymph nodes and T‐cell progenitor thymus seeding
Source: EMBO Rep. 2021 Mar 15;22(4):e52196. doi: 10.15252/embr.202052196 (PMC8024898; doi:10.15252/embr.202052196)

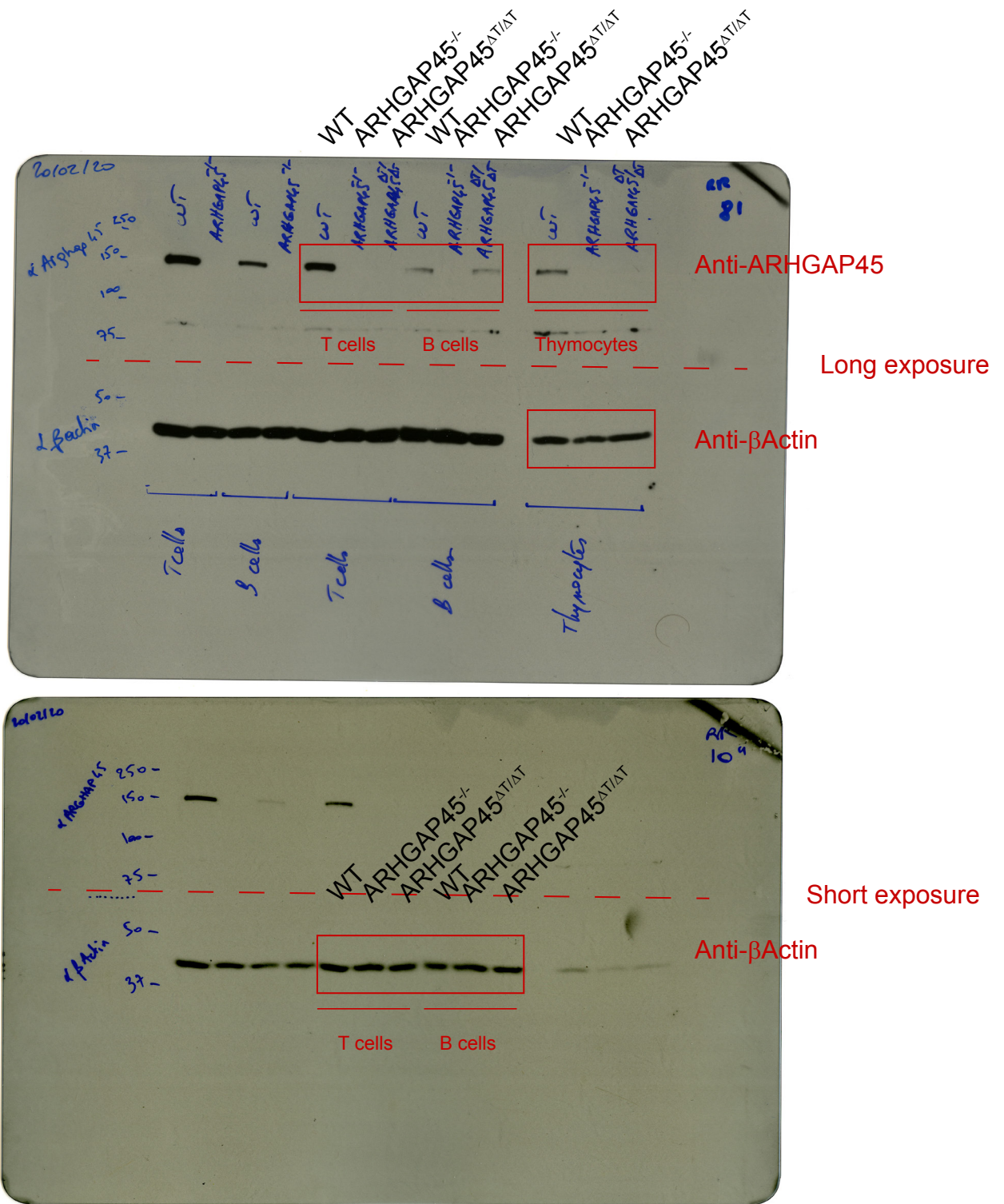

Figure EV1

Supplement: Supplementary file 8 — Source Data for Expanded View [file EMBR-22-e52196-s007.zip › embr202052196-sup-0009-SDataFigEV1.pdf]
