# Supplementary material for: ARHGAP45 controls naïve T‐ and B‐cell entry into lymph nodes and T‐cell progenitor thymus seeding
Source: EMBO Rep. 2021 Mar 15;22(4):e52196. doi: 10.15252/embr.202052196 (PMC8024898; doi:10.15252/embr.202052196)

- To save as much as possible the commercial anti-Arhgap45/HMHA1 sera we bought, the Western blot membrane corresponding to panel A of Figure 1 of our original submission was cutted in two parts. The top part was incubated with the anti-Arhgap45/HMHA1 sera and the bottom part with the anti-GAPDH sera (loading control). For the sake of completion, the corresponding scans of the splitted gel are provided at the end of the present pdf document.
- To be able to provide a scan of an entire gel corresponding to panel A of Figure 1, we repeated the whole experiment in mid-January 2021. The results are totally identical to our previous results and are thus provided in the final version of the manuscript together with the scan of the entire gels (see next two pages).

Total lysates

20210117 ARHGAP45/HMHA1

Jurkat KO12 KI1 KI4 KI1 KI12 KI4

HMHA1

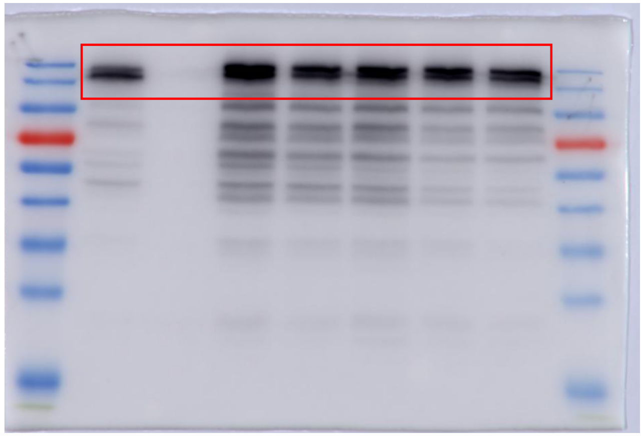

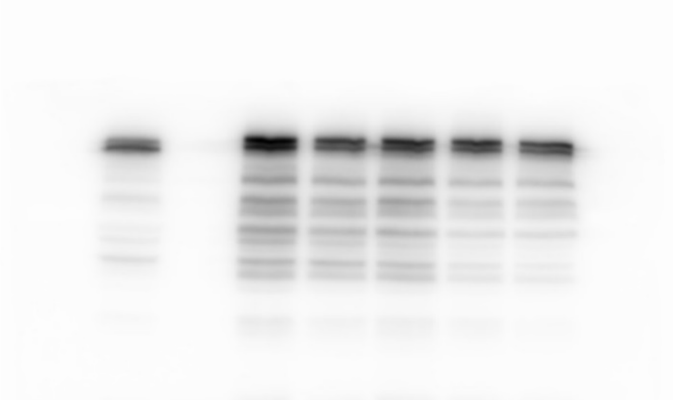

Original image:
4-HAMA1-1 2021.01.18_12.32.27_Ch+Marker

Jurkat KO12 KI1 KI4 KI1 KI12 KI4

GAPDH

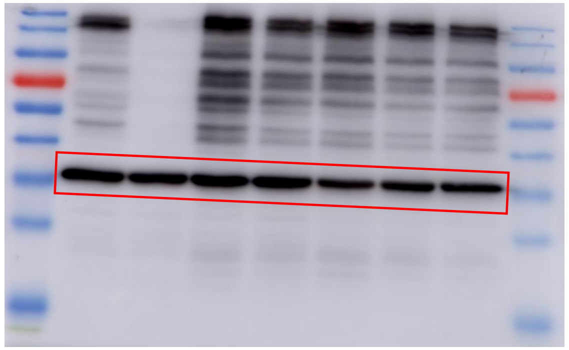

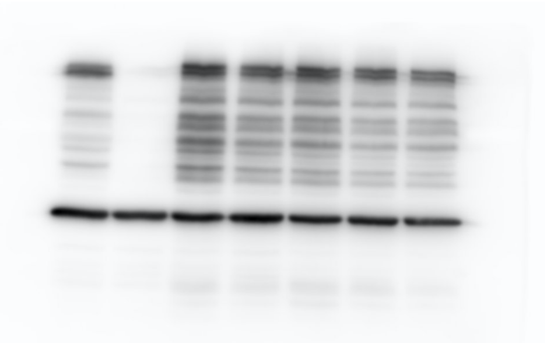

Original image:
4-GAPDH-1 2021.01.18_16.01.23_Ch+Marker

Marker： Thermo 26616, Lot#0961587
antibody：**HMHA1** Sigma HPA019816, Lot#A113728 (1:1000 Rabbit)

20210117 ARHGAP45/HMHA1

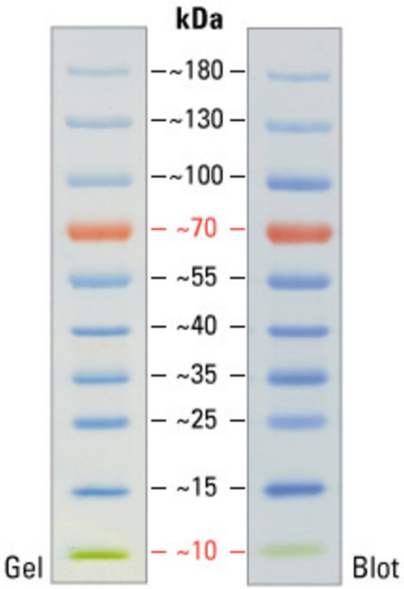

HMHA1

jurkat KO12 KI1 KI4 KI1 KI12 KI4

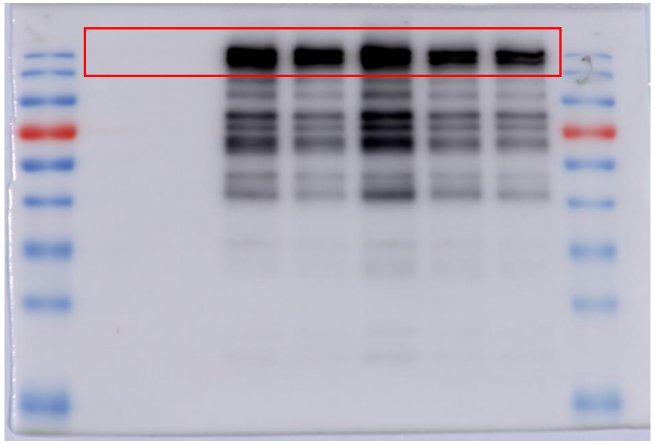

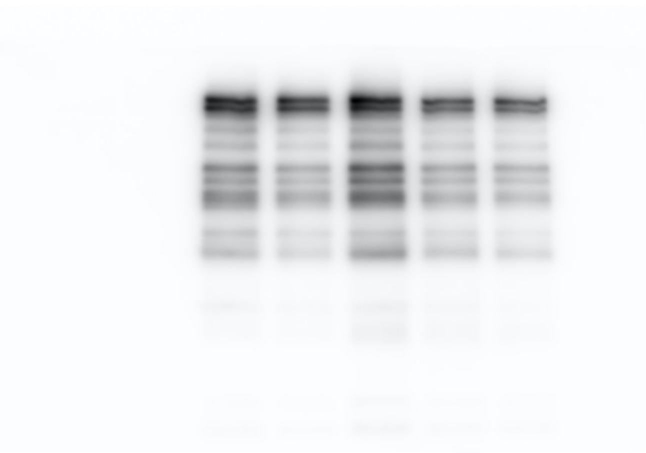

Original image:
2-ip-hama1-2 2021.01.17_21.15.14_Ch+Marker

Marker： Thermo 26616, Lot#0961587
antibody：**HMHA1** Sigma HPA019816, Lot#A113728 (1:1000 Rabbit)

Affinity purification

Material corresponding to original submission

ARHGAP45/HMHA1-20181122

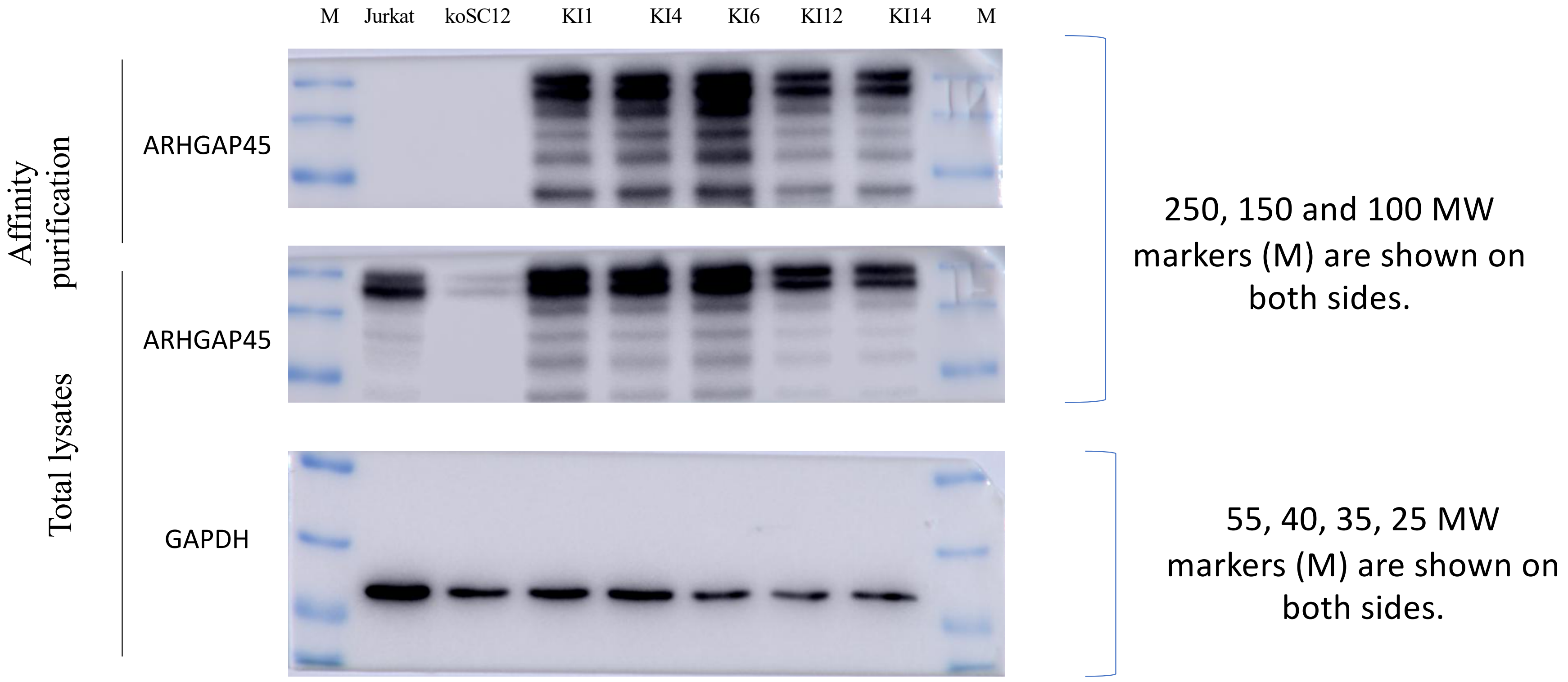

Supplement: Supplementary file 10 — Source Data for Figure 1 [file EMBR-22-e52196-s006.pdf]
